# A Recurrent Pathogenic Variant of *INPP5K* Underlies Autosomal Recessive Congenital Muscular Dystrophy With Cataracts and Intellectual Disability: Evidence for a Founder Effect in Southern Italy

**DOI:** 10.3389/fgene.2020.565868

**Published:** 2020-09-18

**Authors:** Adele D’Amico, Fabiana Fattori, Francesco Nicita, Sabina Barresi, Giorgio Tasca, Margherita Verardo, Simone Pizzi, Isabella Moroni, Francesca De Mitri, Annalia Frongia, Marika Pane, Eugenio Mercuri, Marco Tartaglia, Enrico Bertini

**Affiliations:** ^1^Unit of Neuromuscular and Neurodegenerative Disorders, Department of Neurosciences, Bambino Gesù Children’s Hospital, IRCCS, Rome, Italy; ^2^Genetics and Rare Diseases Research Division, Ospedale Pediatrico Bambino Gesù, IRCCS, Rome, Italy; ^3^Unità Operativa Complessa di Neurologia, Fondazione Policlinico Universitario A. Gemelli IRCCS, Rome, Italy; ^4^Child Neurology Unit, Foundation IRCCS Neurological Institute “C. Besta”, Milan, Italy; ^5^Pediatric Neurology Unit, Catholic University and Nemo Center, Rome, Italy

**Keywords:** INPP5K, CMD, LGMD, cataract, short stature

## Abstract

Inositol polyphosphate-5-phosphatase K [*INPP5K* (MIM: 607875)] acts as a PIP_3_ 5-phosphatase and regulates actin cytoskeleton, insulin, and cell migration. Biallelic pathogenic variants in *INPP5K* have recently been reported in patients affected by a form of muscular dystrophy with childhood onset. Affected patients have limb girdle muscle weakness, often associated with bilateral cataracts, short stature, and intellectual disability. Here we report four patients affected by *INPP5K*-related muscle dystrophy, who were apparently unrelated but originated from the same geographical area in South Italy. These patients manifest a recognizable phenotype characterized by early onset muscular dystrophy associated with short stature and intellectual disability. All affected subjects were homozygous or compound heterozygous for the c.67G > A (p.Val23Met) missense change and shared a common haplotype, indicating the occurrence of a founder effect.

## Introduction

Inositol polyphosphate-5-phosphatase K (*INPP5K*), also known as skeletal muscle and kidney-enriched inositol 5-phosphatase (*SKIP*), is an enzyme that specifically removes the phosphate at position 5 of inositol rings, functioning as a negative regulator of PI3K-AKT signaling (Dong et al., 2018; Berridge and Irvine, 1989). The protein is highly expressed in the developing and adult brain, as well as in eye, muscle, and kidney (Ijuin et al., 2000). Biallelic variants impairing catalytic activity of *INPP5K* have causally been associated with a form of congenital muscular dystrophy characterized by cataracts, cerebellar atrophy, epilepsy, intellectual disability, and short stature (Osborn et al., 2017; Wiessner et al., 2017). The first two series of patients were reported in 2017. Osborn and collaborators (Osborn et al., 2017) described five affected individuals from four unrelated families, presenting variable clinical features, including short stature, intellectual disability, and cataracts. Notably, two unrelated cases of this series, both originated from Italy, were found to be homozygous or compound heterozygous for the c.67G > A; p.Val23Met missense change. Additional patients from families originated from Pakistan and Bangladesh were soon thereafter reported (Wiessner et al., 2017; Yousaf et al., 2018). These patients had a similar phenotype with quite a variability in the age at onset and presenting symptoms, ranging from early presentation with congenital cataracts and/or early motor delay to a later onset after the first years. An additional patient with biallelic inactivating *INPP5K* variants, who was reported to have an uncharacterized syndromic short stature, has more recently been identified by whole exome sequencing (WES) (Homma et al., 2019).

Here we report on four additional patients affected by *INPP5K*-related muscle dystrophy. All affected subjects, who were apparently unrelated but originated from the same geographical area in southern Italy, were homozygous or compound heterozygous for the previously reported c.67G > A; p.Val23Met missense variant, and all share a common haplotype, suggesting a founder effect of this variant in the population of South Italy.

## Materials and Methods

### Patients

Four individuals from apparently unrelated families originating from different little towns (<5,000 inhabitants) of Southern Italy in the province of Lecce (Puglia region) were included in the study. Clinical data and biological material collection, analysis, and storage were obtained from each participating family after obtaining signed informed consent. The study was conducted in accordance with the Declaration of Helsinki. All patients and/or parents of minors signed consent forms for participation to this research observational study and for data publication.

### Clinical Data

Demographic and clinical data of patients, including age at onset, motor outcomes, muscle and brain MRI findings, laboratory results, and muscle histology were collected for all patients and summarized in Table 1.

**TABLE 1:** Clinical features of patients with *INPP5K*^*V23M*^ variant.

Patient	#1	#2	#3	#4	#5	#6
Protein change	p.Val23Met p.Val23Met	p.Val23Met p.Val23Met	p.Val23Met p.Leu55Phe	p.Val23Met p.Gly424Trp	p.Val23Met p.Val23Met Osborn et al., 2017	p.Val23Met p.Asp269Asn Osborn et al., 2017
Gender	F	M	F	F	M	F
Age at onset (years)	6	1	1	2	2	2
Current age (years)	27	18	10	38	24	34
Short stature	+	+	+	+	+	+
Intellectual disability	+	+	+	+	+	+
Microcephaly	+	+	+	+	+	–
Cataracts	–	+	+	+	–	+
Muscle biopsy	Dystrophic	Myopatic changes	Dystrophic	Myopatic changes	Dystrophic	Dystrophic
Mobility	Wheelchair bound, 24 ys	Ambulant	Ambulant	Wheelchair bound, 26 ys	Wheelchair bound, 28 ys	Wheelchair bound, 12 ys
Epilepsy	–	–	+	–	–	–
Brain MRI		Cerebellar atrophy, 12 ys		Cerebral and cerebellar atrophy, 26 ys	Cerebral atrophy, 18 ys	
CK	580 UI/l	700 UI/l	1400 UI/l	500 UI/l	>1,000 UI/l	Elevated

**ys, years; CK, creatinkinase.**

### Muscle Biopsy

Open muscle biopsies of the quadriceps femoris were performed for diagnostic purposes. Specimens were frozen in propane or isopentane and chilled by liquid nitrogen for histochemical analysis. Cryostat muscle sections were stained with hematoxylin and eosin and Gomori Trichrome with standard histological and histochemical protocols.

### Genetic Analysis

#### Next-Generation Sequencing Analyses

Genomic DNA from probands and their parents was extracted from peripheral blood according to manufacturer’s protocol (Qiagen).

In patient #1, exome capture was carried out using the SureSelect Human All Exon V4 (Agilent) enrichment kit, and parallel sequencing was performed using an Illumina HiSeq 2000 platform. WES data analysis was performed using an in-house implemented pipeline, which mainly take advantage of the Genome Analysis Toolkit (GATK V.3.7) (McKenna, 2009) framework, as previously reported (Flex et al., 2016; Bauer et al., 2018). Functional annotation of variants was achieved using SnpEff and dbNSFP (V.3.0) (Dong et al., 2015), and their functional impact was assessed by Combined Annotation Dependent Depletion (CADD) V.1.3, M-CAP V.1.0, and InterVar V.0.1.6 (Kircher et al., 2014; Jagadeesh et al., 2016; Li and Wang, 2017).

In patients #2, #3, and #4, a mutation scan of the entire coding sequence of *INPP5K* was performed by parallel sequencing using a customized gene panel opportunely designed to include 121 muscular-related disease genes. Target enrichment was carried out using Nextera Rapid Capture Custom Enrichment Kit (Illumina, San Diego, California, United States) with probe design performed by using the Design Studio software. Paired-end sequencing was performed using an Illumina MiSeq with a sequencing depth of 100×. The Illumina VariantStudio data analysis software was used to annotate the variants.

#### Haplotype Analysis

The four patients of our cohort and the two Italian *INPP5K*^*V*23*M*^ cases already described (Osborn et al., 2017), together with their parents, were genotyped for single nucleotide polymorphisms (SNPs) and/or short tandem repeats (STRs) opportunely selected within the homozygous genomic region encompassing *INPP5K* and spanning 1.6 Mb, that had been identified in WES data analysis of Pt #1 by using Homozygosity mapper tool (Seelow et al., 2009). For the determination of the shared haplotype, the analyzed genomic regions containing known SNPs (rs118109021, rs3752827, rs8072859, rs8069059, rs8079811, rs1059139, rs2240999, rs34308535, rs398030149, rs3815465, rs2277669, rs7342891, and rs12953268) were amplified by polymerase chain reaction (PCR) from genomic DNA, and the purified PCR products were sequenced by Sanger sequencing. Amplification of a selected panel of STRs (D17S1174, D17S1510E, D17S1376E, D17S1577, and D17S1533) was performed using 5’-FAM–labeled primers, according to standard procedures. Fragment analysis was performed on an ABI 3130xl capillary sequencer using GeneMapper software (Applied Biosystems, Foster City, CA).

## Results

### Clinical Data

**Patient #1** is a 27-year-old female born as the second child from parents with not declared consanguinity, originated from a small area of South Italy (Puglia). First motor milestones were regularly achieved, whereas cognitive delay was observed since early infancy. By the age of 10 years, she started to present progressive proximal muscle weakness mainly involving lower limbs. Our first neuromuscular examination was performed at the age of 22 years showing microcephaly and short stature (below the third percentile), severe hyperlordosis, pseudohypertrophy of calves, and hypotrophy of quadriceps. The patient also had weakness in upper limbs, and she was unable to raise arms to the shoulders (MRC score of deltoids and brachial biceps was 3). She was able to walk for 20 m. Creatinkinase (CK) was elevated up to two times the normal values (500 UI/l, n.v. <270). Moreover, a moderate intellectual disability was diagnosed, whereas ophthalmological examination did not reveal cataracts. Muscle weakness progressively worsened, and the patient lost the ambulation at the age of 24 years, as well as upper limbs muscle strength (last MRC scores were 2 for deltoids, 3 for biceps brachialis). Brain MRI performed at the age of 22 years, did not show cerebellar atrophy.

**Patient #2** is an 18-year-old boy. He is the only child of non-consanguineous parents, both originating from Puglia. By early infancy, he presented with psychomotor delay and acquired autonomous ambulation at the age of 2 years. At 3 years of age, he underwent surgery for bilateral congenital cataracts. At the age of 4 years, short stature (below the third percentile), microcephaly, ataxic gait, and mild weakness of limb girdle muscles with a positive Gower’s sign were observed at first neurological examination; CK was elevated up to roughly three times the normal values. In the following years, muscle weakness progressively worsened. At the last clinical examination, at the age of 16 years, the patient had ataxic and waddling gait, and he was unable to rise from the floor and to climb a step. He also had scapular winging and weakness of deltoids (MRC score = 4). Brain MRI performed at the age of 12 years showed mild cerebellar atrophy.

**Patient #3** is a 10-year-old girl. She is the only child of unrelated parents both originated from Puglia. First investigation started at the age of 3 years because she always had growth retardation (height and weight below the third percentile). Extensive metabolic end endocrinological was negative as well as investigation for celiac disease. Following the occasional finding of high CK levels (up to 1400 UI/l, n.v. <250) she underwent neurological examination that did not reveal muscle weakness or fatigability. However, because the baby had a speech delay, a cognitive assessment was performed, and a diagnosis of mild intellectual disability was formulated. Afterward, she manifested epileptic crisis suggestive of Panayiotopoulos epilepsy, and pharmacological treatment was started. Brain MRI was normal. Muscle MRI and muscle biopsy were performed at the age of 5 years. The muscle MRI did not reveal abnormalities in T1-weigh images, whereas dystrophic features were documented at muscle biopsy. At present, the baby has difficulty in running, jumping, and in raising her arms above the shoulders.

**Patient #4** is a 38-year-old woman. She is the first child of three siblings who did not show signs of muscular disease or intellectual disability. The unrelated parent originated from the same geographic area of other patients (Puglia). By early infancy, she underwent several investigations for growth delay (height and weight always below the third percentile) and a high level of CK was occasionally found at the age of 2 years. At the age of 3 years, she underwent surgery for bilateral congenital cataracts. She acquired the autonomous ambulation at 18 months, but she always walked on toes and had a waddling gait. At the age of 8 years, a muscle biopsy was performed showing dystrophic features. Over the following years, an intellectual disability was diagnosed, and she also worsened in motor abilities. Our first neurological examination was performed at the age of 36 years. The patient has scoliosis, microcephaly, and short stature (below the third percentile). She presented diffuse muscle wasting and weakness and ankle contractures. She was able to raise her arms above her head (deltoids and brachial biceps MRC = 4) and able to stand without support, but she used a wheelchair for nearly two years for mobility.

### Muscle Biopsy

All patients underwent a muscle biopsy for diagnostic purposes at the ages of 22 years (#1), 5 years (#2), 4 years (#3), and 11 years (#4), respectively.

In patients #1 and #3, a histological examination showed marked fiber diameter, increased connective tissue, centrally located myonuclei, and scattered necrotic fibers.

In patients 2# and 4#, unspecific myopathic changes consisted of centrally located nuclei and fiber size variation.

### Muscle Imaging

A muscle MRI was also performed in all patients at the age of 22 years (#1), 12 years (#2), 5 years (#3), and 36 years (#4), respectively.

**FIGURE 1:**
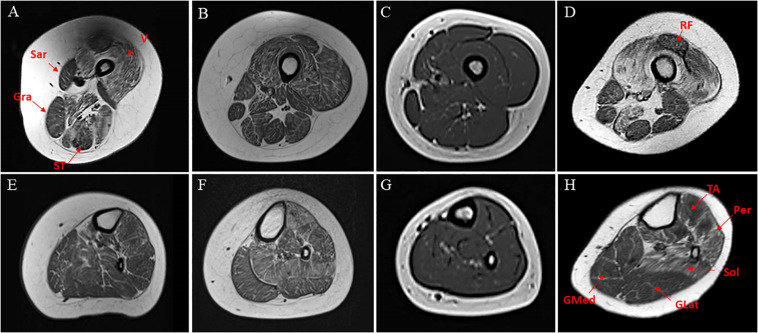
Transverse T1-weighted MRI of thighs and calf muscles of patients harboring INPP5K mutations. Pt #1 **(A,D)**, Pt #2 **(B,E)**, Pt #3 **(C,F)**, pt #4 **(D,G)**. At thigh level, an involvement of vasti muscles (V) are relative sparing of rectus femoris (RF) in patients #1and #4 **(A,D)**. In the posterior thigh, sartorius (Sar), gracilis (Gra), and to a lesser extent, semitendinosus (ST) were relatively spared **(E,H)**. At the lower leg level, anterolateral compartment (tibialis anterior-T ant- and peronei –Per-) and soleus (Sol) were similarly affected, with relative sparing of both gastrocnemii (GMed and GLat) muscles mainly in #4 **(H)**. In the youngest patient, the MRI did not disclose significant abnormalities **(C,G)** on T1-weighted images.

In patients #1 and #4, at thigh level, T1-weight images revealed a consistent involvement of vasti muscles with relative sparing of rectus femoris. Sartorius, gracilis, and, to a lesser extent, semitendinosus were selectively spared, whereas adductors magnus were severely affected. At the lower leg level, the anterolateral compartment (peronei in particular) and soleus were similarly affected, with relative sparing of both gastrocnemii muscles. Patient #3 had a similar but milder pattern of involvement, whereas in patient #4 the MRI did not show any significant abnormalities (Figure 1).

Clinical findings are summarized in Table 1.

The two patients originally reported by Osborn and colleague (Osborn et al., 2017) harboring p.Val23Met variant have been also included in Table 1 (patients #5 and #6) to provide a more accurate profile of the clinical phenotype associated with this variant. The families of these two additional patients are also originated from the same geographical area of South Italy, and we extended the haplotype analyses in these two pedigrees.

### Molecular Analyses

WES and targeted sequencing performed in the diagnostic setting lead us to understand the molecular basis of an unclassified muscular dystrophy disorder in four apparently non-related subjects aged between 10 and 38 years from southern Italy and allowed to identify biallelic variants in *INPP5K* as the underlying cause. In patient #1, WES data analysis identified 50.567 high-quality non-synonymous and splice site variants. Among them, 204 variants were private, rare, or clinically associated. Parental inbreeding was not declared by parents of patient #1, although a large region of homozygosity were found in this patient, suggesting a degree of consanguinity between parents. Taking into account homozygosity by descent, filtering of variants predicted to have a disruptive impact on protein function allowed to identify the c.67G > A missense substitution, p.Val23Met, in exon 2 of *INPP5K* gene as the only excellent candidate event underlying the trait. Based on the informative variants flanking the pathogenic variant, the homozygous genomic region encompassing the c.67G > A variant was estimated to cover approximately 1.6 Mb (chr17: 6157- 1613203). In patients #2, #3, and #4, parallel sequencing using a customized gene panel for congenital myopathies revealed the same missense c.67G > A variant in *INPP5K*, which was found in the homozygous state in patient #2 and in compound heterozygosity with two distinct novel missense variants, c.165G > T (p.Leu55Phe) and c.1270G > T (p.Val424Trp) in patients #3 and #4, respectively (Table 2). Variant validation in probands and segregation analysis in their unaffected parents were confirmed by Sanger sequencing.

**TABLE 2:** Summary of pathogenic variants identified in this study.

Pt	Ethnic origin	Genotype	cDNA	Protein	gnomAD (allele frequency)	NM_transcript	Protein domain	References
Pt 1	Italy (Puglia)	Homo	c.67G > A c.67G > A	p.(Val23Met) p.(Val23Met)	0.0008% 0.0008%	NM_016532.4	Phosphatase	Osborn et al., 2017
Pt 2	Italy (Puglia)	Homo	c.67G > A c.67G > A	p.(Val23Met) p.(Val23Met)	0.0008% 0.0008%	NM_016532.4	Phosphatase	Osborn et al., 2017
Pt 3	Italy (Puglia)	Het Het	c.67G > A c.165G > T	p.(Val23Met) p.(Leu55Phe)	0.0008% NA	NM_016532.4	Phosphatase Phosphatase	Osborn et al., 2017 This study
Pt 4	Italy (Puglia)	Het Het	c.67G > A c.1270G > T	p.(Val23Met) p.(Val424Trp)	0.0008% NA	NM_016532.4	Phosphatase Actin ruffle targeting	Osborn et al., 2017 This study

**NA, not available.**

The two novel c.165G > T and c.1270G > T variants, which affect highly conserved residues in the phosphatase domain and in the actin ruffle targeting domain, respectively, are not present in gnomAD or other databases, were predicted to be deleterious by M-CAP, and obtained an overall CADD score of 22.9 and 27.6, respectively.

The *INPP5K* gene has been screened, through our customized next-generation sequencing (NGS) gene panel reported in more than 300 Italian patients from all over Italy affected by muscular disease. The c.67G > A, c.165G > T, and c.1270G > T variants have never been found even in heterozygous trait in screened patients thus confirming the very low frequency of these variants.

The identification of the same variant in these four unrelated patients and in the two Italian cases previously reported by Osborn and collaborators (Osborn et al., 2017), who were from apparently unrelated families from the same area of southern Italy, suggested a founder event for the c.67G > A variant.

**FIGURE 2:**
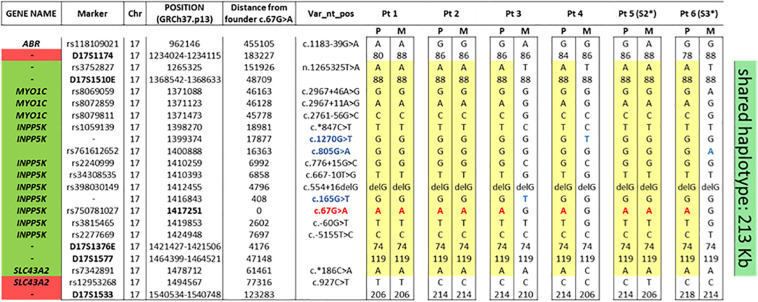
The common haplotype showing the minimal common region among the six patients. As can be seen, the nine alleles with the *INPP5K* c.67G > A variant (in red) shared a common region between rs3752827 and rs7342891 (213 kb). In blue, the other three *INPP5K* variants in compound heterozygosis with the founder c.67G > A variant. Segregation analysis in both parents of the six patients allowed to reconstruct parental genotype. *P*, paternal allele; *M*, maternal allele. * Patient already reported by Osborn et al. (2017).

Haplotype analysis, performed in probands and in their unaffected parents, using SNPs and STR markers selected within a 1.6 Mb homozygous region surrounding the *INPP5K* c.67G > A variant revealed by WES data in Pt #1, allowed to identify a shared haplotype encompassing 15 consecutive SNPs/STRs spanning a 213 kb region delimitated by markers rs3752827 and rs7342891 (chromosome 17:1265325–1478712) (Figure 2). This common haplotype is also shared by the two Italian patients originally described by Osborn in 2017 (Osborn et al., 2017) that have been included in this study.

This common haplotype strongly supports a founder effect for this pathogenic variant.

## Discussion

The *INPP5K* gene encodes inositol polyphosphate-5-phosphatase K, an enzyme, highly expressed in the developing and adult brain, eye, muscle, and kidney (Ijuin et al., 2000), that catalyzes the dephosphorylation of the phosphate of the inositol ring on position 5, thereby acting as a negative regulator of PI3K intracellular signaling. Also known as *SKIP*, *INPP5K*, which has a simple two-domain structure with an N-terminal 5-phosphatase domain followed by a C-terminal SKICH domain, regulates the actin cytoskeleton, myoblast differentiation, and insulin signaling in skeletal muscle (Ijuin et al., 2000, 2008; Osborn et al., 2017) and has been reported to be involved in the fine control of endoplasmic reticulum (ER) network organization (Dong et al., 2018).

Biallelic point variants that impair the phosphatase activity of *INPP5K*, giving rise to excess PtdIns(4,5)P_2_ in affected individuals’ cells, have been related to congenital muscular dystrophy with additional clinical manifestations, including cataracts, intellectual impairments, and short stature (Osborn et al., 2017; Wiessner et al., 2017). These variants likely only result in partial loss of *INPP5K* function because complete loss of *INPP5K* expression in mice results in embryonic lethality (Ijuin et al., 2008).

In this study, we describe four patients affected by a form of muscular dystrophy related to a recurrent c.67G > A (p.Val23Met) amino acid substitution in *INPP5K*. The very rare c.67G > A variant is reported in gnomAD database only in two heterozygous carriers (out of 250,252 allele screened) from European non-Finnish population confirming for biallelic loss being necessary for disease. Moreover, in our cohort of more than 300 Italian patients from all over Italy affected by muscular disease, this variant has never been found, even in heterozygous trait, thus confirming the very low frequency of this variant also in the Italian population.

The four patients in our cohort and the two previously described are all unrelated patients from a small area of Puglia, a region in Southern Italy. All patients had some signs of muscle weakness and/or motor delay in early infancy, with variable degree of progression, and mainly involving the proximal muscles. Some patients lost the ability to walk in the second decade while others were still ambulant in the third decade. Mild intellectual disability and short stature were invariably present in all. Cataract (4/6), epilepsy (1/6), and microcephaly (5/6) were also observed in some of them.

Cerebral and cerebellar atrophy were also documented in two patients (as also reported in one of the two Italian patients reported by Osborn, patient #5).

Muscle MRI showed consistent involvement of vasti, anterior tibialis and peronei muscles, and the degree of muscle degeneration appears to be age-related being more severe in older patients (Figure 1).

Muscle biopsies performed in all patients showed myopathic findings in patient #2 and patient #4 and variable dystrophic features in patients #1 and #3.

In our opinion, patients harboring the pVal23Met variant have a homogeneous and easily recognizable phenotype synthesized in the triade of intellectual disability, muscular dystrophy, and short stature. Muscle imaging findings support this homogeneity, showing a similar pattern in three patients with different degrees of severity. Epilepsy, brain atrophy, and cataracts were additional features not observed in all patients. Intellectual disability in these patients is probably consistent with a role of *INPP5K* in neurons (Fink et al., 2017; Osborn et al., 2017; Wiessner et al., 2017). In fact, *INPP5K* was reported to be upregulated in regenerating mammalian spinal cord axons, and overexpression of this gene has been described in acutely dissociated mouse cortical neurons enhanced neurite outgrowth and branching during the recovery after *in vitro* injury (Fink et al., 2017). In both our series and in the previously reported cases, patients had onset of clinical signs in the first years that in some cases could be consistent with a diagnosis of congenital muscular dystrophy, while others had onset well after the children had acquired the ability to walk and would be more compatible with a diagnosis of limb girdle muscle dystrophy (LGMD). Even, in our series, we observed a phenotypic variability also in those patients who has the same homozygous Val23Met variant. This finding underlies that the mechanisms of the disease still remain unknown. As observed in other forms of muscular dystrophies, mutations in the same gene can produce a broad range of clinical phenotypes ranging from CMD to LGMD with variable involvement of central nervous system (Bertini et al., 2011). It has been demonstrated that *INPP5K* participates with the ER function and organization. However, the other genetic and epigenetic factors that certainly contribute to ER function may have a role in the expression of the disease and phenotypic variability.

To investigate whether a founder effect rather than a mutation hot-spot phenomenon was involved, haplotype sharing analyses were performed for c.67G > A variant, showing a common founder. In fact, all nine alleles with this variant in the four patients of our cohort and in the two Italian cases already described by Osborn et al. (2017) shared a common haplotype composed of 15 markers around this disease locus sharing a haplotype region of 213 kb. Founder mutations have been proven to impact upon molecular diagnosis strategies in specific populations. Despite current availability of NGS, in the presence of some clinical features easily attributable to mutations in specific genes, founder mutations can be assessed as the first screening step of candidate gene approach, and if positive, further time-consuming gene scanning can be avoided. In fact, our findings of a founder effect for the *INPP5K* c.67G > A variant in six unrelated Italian patients affected by an easy recognizable phenotype, characterized by the triade of muscle dystrophy, short stature, and intellectual disability and should lead us to consider for the diagnosis of this clinical phenotype, especially if originating from the same area of southern Italy (Puglia), sequencing of exon 2 of *INPP5K* as the first screening step.

In summary, this is the first study that describes the founder effect of the *INPP5K* c.67G > A variant in southern Italy. All patients manifest recognizable phenotype characterized by muscular dystrophy associate to short stature and intellectual disability. Further studies are needed to confirm this hypothesis and to estimate with sufficient confidence the age of mutation. Moreover, we are reporting two additional novel variants in *INPP5K* expanding the mutational spectrum of this gene.

## Data Availability Statement

The datasets for this article are not publicly available due to concerns regarding participant/patient anonymity. Requests to access the datasets should be directed to the corresponding author.

## Ethics Statement

All patients and/or parents of minors signed consent forms for participation to this research observational study and for data publication.

## Author Contributions

AD’A, FF, MT, and SB conceived and planned the experiments. FF, FD, and SB carried out the experiments. MT, SP, AD’A, and FF contributed to the interpretation of the results. GT revised musle MRI. IM, MP, AF, and FN contributed to clinical data collection. MV set up muscle biopsies. AD’A took the lead in writing the manuscript. EB, EM, and MT revised the manuscript. All authors provided critical feedback and helped shape the research, analysis and manuscript.

## Conflict of Interest

The authors declare that the research was conducted in the absence of any commercial or financial relationships that could be construed as a potential conflict of interest.
